# The chimeric monoclonal antibody MHCSZ-123 against human von Willebrand factor A3 domain inhibits high-shear arterial thrombosis in a Rhesus monkey model

**DOI:** 10.1186/s13045-017-0475-2

**Published:** 2017-05-19

**Authors:** Shundong Ji, Miao Jiang, Bin Yan, Fei Shen, Yang He, Aini Wan, Lijun Xia, Changgeng Ruan, Yiming Zhao

**Affiliations:** 1 The First Affiliated Hospital of Soochow University, Jiangsu Institute of Hematology, MOH Key Laboratory of Thrombosis and Hemostasis, Collaborative Innovation Center of Hematology, 188 Shizi Street, Suzhou, Jiangsu 215006 China; 2 MOE Key Laboratory of Industrial Biotechnology, School of Biotechnology of Jiangnan University, 1800 Lihu Avenue, Wuxi, Jiangsu 214122 China; 3 Cardiovascular Biology Research Program, Oklahoma Medical Research Foundation, Oklahoma City, OK 73104 USA

**Keywords:** Thrombosis, Collagen, Von Willebrand factor (VWF), Platelet, Platelet aggregation, Monoclonal antibody

## Abstract

**Background:**

SZ-123, a murine monoclonal antibody that targets the human von Willebrand factor (VWF) A3 domain and blocks the binding of collagen, is a powerful antithrombotic. In a Rhesus monkey model of thrombosis, SZ-123 had no side effects, such as bleeding or thrombocytopenia.

**Methods:**

The mouse/human chimeric version of SZ-123, MHCSZ-123, was developed and maintained inhibitory capacities in vitro and ex vivo after injection into monkeys. CHO-S cells were selected for stable expression of MHCSZ-123. Cell clones with high levels of MHCSZ-123 expression were screened with G418 then adapted to serum-free suspension culture. The antithrombotic effect of MHCSZ-123 on acute platelet-mediated thrombosis was studied in monkeys where thrombus formation was induced by injury and stenosis of the femoral artery, which allowed for cyclic flow reductions (CFRs). CFRs were measured in the femoral artery of anesthetized Rhesus monkeys before and after intravenous administration of MHCSZ-123. Ex vivo VWF binding to collagen, platelet aggregation, platelet counts, and template bleeding time were used as measurements of antithrombotic activity. In addition, plasma VWF and VWF occupancy were measured by ELISA.

**Results:**

Injection of 0.1, 0.3, and 0.6 mg/kg MHCSZ-123 significantly reduced CFRs by 29.4%, 57.9%, and 73.1%, respectively. When 0.3 and 0.6 mg/kg MHCSZ-123 were administered, 46.6%–65.8% inhibition of ristocetin-induced platelet aggregation was observed between 15 and 30 min after injection. We observed minimal effects on bleeding time, minimal blood loss, and no spontaneous bleeding or thrombocytopenia.

**Conclusions:**

The VWF-A3 inhibitor MHCSZ-123 significantly reduced thrombosis in Rhesus monkeys and appeared to be safe and well tolerated.

## Background

Platelets are the key players in primary hemostasis. The interaction between von Willebrand factor (VWF), and platelet glycoprotein (GP) Ibα is the key for initiating the response to vascular injury that leads to hemostasis or, under pathological conditions, thrombosis [1–3]. VWF binds to exposed collagen matrix through its A3 domain. After binding, it undergoes conformational changes that allow it to bind to the platelet receptor GPIbα through the A1 domain, thereby mediating platelet adhesion to the subendothelium [2, 3]. The binding of VWF to GPIbα and its role in platelet adhesion and aggregation become progressively more important with increasing shear rates, i.e., in stenotic coronary arteries or ruptured atherosclerotic plaque lesions [3].

Many different components have been identified that contribute to thrombosis, and several therapeutic approaches have been developed for this severe clinical complication. While these treatments have been effective in the clinical setting, they have a limited therapeutic window and are associated with clinically significant bleeding, particularly when high doses of the drugs are used [4, 5]. In the last several years, a number of new agents have been under (pre) clinical investigations that target the GPIbα- VWF-collagen axis, including GPIb-binding snake venom proteins [6], anti-GPIb antibodies [7, 8], anti-VWF A1 domain monoclonal antibodies (mAbs) [9–11], and anti-VWF A3 domain mAbs [12, 13]. In addition, non-antibody-based approaches have been developed, including the VWF-specific aptamer ARC1779 [14, 15] and soluble GPIbα fragments [16, 17]. VWF exemplifies the thin line between normal hemostasis and an overly active system that promotes thrombosis [18]. Its levels are also heightened in patients, who experienced adverse cardiac events that are linked to a poorer prognosis [19–21].

Previously, we prepared and characterized a murine monoclonal antibody (mAb) against the human VWF A3 domain, designated SZ-123. SZ-123 specifically blocked binding of purified human VWF to collagen type III and prevented the VWF-dependent adhesion of human platelets to collagen under high shear stress [22]. We demonstrated that SZ-123 prevented arterial thrombus formation under high shear conditions by inhibiting VWF A3 collagen interaction in Rhesus monkeys [23]. In addition, SZ-123 also masked the binding site of VWF A1 to GPIbα and, therefore, indirectly inhibited the interaction between VWF A1 and GPIbα [23].

As a further step in the development of SZ-123, we constructed a mouse/human chimeric antibody derived from hybridomas producing murine antibody against the human VWF A3 domain and induced its expression in Chinese hamster ovary (CHO-S) cells. The chimeric antibodies consisted of variable regions from the mouse antibody and constant regions from the human antibody. This type of antibody eliminates the highly immunogenic murine constant region while maintaining its antigen binding affinity [24]. The mouse/human chimeric antibody SZ-123 (named MHCSZ-123) specifically inhibited VWF-mediated platelet adhesion, aggregation, and activation at concentrations comparable to the parent monoclonal antibody. The objectives of this study were to validate the antithrombotic properties of MHCSZ-123 against high-shear arterial thrombosis in a Rhesus monkey model.

## Methods

### Reagents

Blood donations were from healthy volunteers who provided written informed consent. The pMH3 vector and CHO-S cells were purchased from AmProtein (Hangzhou, China). Restriction enzymes, T4 DNA ligase, and Taq DNA polymerase were obtained from New England BioLabs (Beverly, MA, USA). Rabbit anti-human VWF polyclonal antibody and rabbit anti-human VWF-HRP antibody were purchased from Dako Cytomation (Glostrup, Denmark). Ristocetin, tetramethylbenizidine (TMB), and collagen type III from human placenta were purchased from Sigma (St. Louis, MO, USA). Goat anti-mouse IgG and goat anti-mouse IgG labeled with horseradish peroxidase (HRP) were purchased from Beckman-Coulter (Brea, CA, USA). SZ-123, a murine anti-human VWF A3 domain mAb (IgG1) was produced by standard hybridoma technology in our laboratory as described previously [22]. SZ-34, a murine mAb that binds to the VWF A2 domain but does not inhibit its function, was produced by standard hybridoma technology in our laboratory [25].

### RNA extraction and 5’RACE

Complementary DNAs (cDNAs) for the variable gene of the heavy and light chains of SZ-123 were amplified using the 5’ rapid amplification of cDNA end (5’RACE) method. Briefly, total RNA was isolated from an SZ-123 monoclonal antibody-expressing hybridoma with TRIzol reagent according to the manufacturer’s protocol. The 5’RACE method was performed according to the protocol provided by the manufacturer (Takara, Tokyo, Japan). The gene-specific primers HGSP1 and KGSP1 used for reverse transcription are listed in Table 1. After reverse transcription, variable regions and signal peptide sequences of SZ-123 were amplified by PCR with the adaptor primers, HGSP2 and KGSP2, for heavy chain and light chain, respectively, (listed in Table 1). The PCR products were cloned into the pMH3 vector, and inserts were confirmed by DNA sequencing (Shanghai Bioengineering, Shanghai, China).

**Table 1:** Gene-specific primers HGSP1 and KGSP1

HGSP1	GTCCACCTTGGTGCTGCTGGCCGGGTG
KGSP1	ACTTGACATTGATGTCTTTG
HGSP2	GCTGGACAGGGATCCAGAGTTCC
KGSP2	CACGACTGAGGCACCTCCAG

### Construction of the MHCSZ-123 VH and VL expression vectors

The pMH3 vector (AmProtein, Hangzhou, China), a eukaryotic expression vector that includes the chicken beta actin gene intron-1 promoter (GC content average 75.3%, the highest 90.8%), was selected for high expression of the chimeric antibody [26]. pMH3H and pMH3L vectors carried the cDNAs of the IgG1 heavy chain and kappa chain constant regions, respectively, which resulted in GC-rich DNA structures.

To construct the pMH3-MHC-SZ-123VH and pMH3-MHC-SZ-123VL expression vectors, two sets of primer pairs, SZ-123HR, SZ-123HF for VH and SZ-123LR, SZ-123LF for VL, were designed and synthesized based on the sequences of 5’RACE results. Primer sequences are listed in Table 2. PCR conditions were as follows: 94 °C for 4 min followed by 35 cycles of 94 °C for 30 s, 58 °C for 30 s, and 72 °C for 30 s, and a final 10 min at 72 °C for complete extension. The PCR products were separated in a 1% agarose gel, and the target bands were gel purified. After digestion of the PCR products with restriction enzymes (EcoRI and NotI for both VH and VL), the VH and VL cDNAs were cloned directly into pMH3-MHC-SZ-123VH and pMH3-MHC-SZ-123VL, respectively. The cDNA inserts were confirmed by DNA sequencing.

**Table 2:** Gene-specific primers SZ-123VH and SZ-123VK

p-VH-F:	GCGAATTCCACCATGGAGACAGACACACTCCTGCTATGGGTACTG
	CTGCTCTGGGTTCCAGGTTCCACTGGTCAGGTCCAACTGCAGCAG
	CCTGGGGCTGAACTGGTGAAG
p-VH-R:	AGTGGCGGCCGCGGATCCGAATTTTAAGTCATTTA
p-VK-F:	GCGAATTCCACCATGGAGACAGACACACTCCTGCTATGGGTACTGC
p-VK-R:	TGGATCAGTTATCTATGCGGCCGCTAACACTCT

The underlined bases represent the recognition sites for the restriction enzymes EcoRI and NotI

### Generation of stable MHCSZ-123-expressing cells clones

CHO-S cells were cultured in DMEM/F12 medium (Gibco, ThermoFisher Scientific, Waltham, MA, USA) supplemented with 10% FBS (Gibco) to obtain 1.5 × 10^7^ cells per milliliter on the day of transfection. pMH3-MHC-SZ-123VH and pMH3-MHC-SZ-123VL expression vectors were transfected into CHO-S cells using electroporation. Briefly, 3 × 10^6^ CHO-S cells in 200 μL DMEM/F12 medium (Gibco) with 10% FBS, 20 μg of expression vector, and 10 μg salmon sperm DNA were mixed together. Electroporation was performed four times at 500 V, 500 μs using a Gene Pulser (BioRad, Hercules, CA, USA). The transfected CHO-S cells were then cultured under selection in DMEM/F12 medium supplemented with 10% FBS containing 2.4 mg/ml G418 (Merck, Kenilworth, NJ, USA) in 10 cm dishes for 1 week at 37 °C. Surviving cell clones were picked and cultured in 96-well plates for another 7 days. Stably transfected cells were screened under G418 for gene copy-number amplification. Twenty-four hours after replacing the G418-containing medium with serum-free medium, the amount of MHCSZ-123 secreted into the medium was quantified using a sandwich ELISA kit (Corning, ThermoFisher Scientific, Waltham, MA, USA). The high-expression cell clones were picked and used for the second round of selection where 100 cells of each high-expression clone were seeded into 24-well plates and selected again according to protocol described above. Finally, the surviving clones were expanded for suspension cultures and freezing. The cells were then adapted to serum-free suspension medium B001 (AmProtein) containing 2.5 mM L-glutamine (Gibco) on a shaker at 120 rpm in a humidified atmosphere at 37 °C and 8% CO_2_.

### Enzyme-linked immunosorbent assay

MHCSZ-123 concentrations were determined in an ELISA assay as described previously (23). Briefly, MHCSZ-123 was captured by goat anti-human IgG1 polyclonal antibody (10 μg/ml) precoated on an ELISA plate. Supernatants from growing colonies and human IgG1 as standard were added to the wells. After 1 h incubation at 37 °C, bound MHCSZ-123 was detected with horseradish peroxide (HRP)-conjugated goat anti-human IgG kappa antibody (1 μg/ml), followed by color development after addition of 3,3’,5,5’-tetramethylbenzidine (TMB) substrate solution. The reaction was stopped with 1 N H_2_SO_4_ upon incubation for 15 min at 37 °C. The plate was read at 450 nm in a microtiter plate reader (ThermoFisher, Waltham, MA, USA).

### Purification of MHCSZ-123

The chimeric antibody against the VWF A3 domain was purified using affinity chromatography with Protein A-Sepharose resin. Briefly, supernatant was harvested then applied to a Protein A-Sepharose 4B column (GE Healthcare, Piscataway, NJ, USA). The bound MHCSZ-123 was eluted with 0.1 M glycine-HCL (pH 3.0). After dialysis against phosphate-buffered saline (PBS) overnight at 4 °C, the antibody was clarified through a 0.22 μm filter. The final purified MHCSZ-123 was stored in PBS at −80 °C. The protein concentration was estimated using a BCA protein assay reagent (Pierce), with BSA as standard.

### Inhibition of Rhesus monkey VWF binding to human collagen type III by MHCSZ-123

In a previous study, we verified that SZ-123 cross-reacted with Rhesus monkey VWF and efficiently inhibited binding of plasma VWF from Rhesus monkey to human collagen type III [23]. To confirm that MHCSZ-123 could inhibit binding of plasma VWF from Rhesus monkeys to human collagen type III, serial concentrations of MHCSZ-123 (2–128 ng/ml) were added to plasma during incubation. Plasma samples of normal monkeys used for the in vitro assay were ordered from Xishan Zhongke (Suzhou, China). The parent mAb SZ-123 was selected as a positive control, and the anti-VWF A2 mAb SZ-34 was used as a negative control.

Collagen binding assays were performed by ELISA as described [22, 23]. Briefly, 96-well microtiter plates were coated with type III collagen from human placenta and blocked with 2% BSA-PBS. Rhesus monkey plasma was incubated with MHCSZ-123 or control antibodies (SZ-123 and SZ-34). After washing, binding was detected with rabbit anti-human VWF-HRP antibodies combined with TMB. Binding of VWF to collagen at each concentration was compared to the binding of VWF without antibodies, which was set as 100%.

### Ristocetin-induced platelet aggregation

Platelet aggregation assay was performed as described previously [22, 23]. Human platelet suspension was prepared at ~2.5 × 10^5^ per microliter by mixing platelet-rich plasma (PRP) and autologous platelet-poor plasma (PPP) prepared by differential centrifugation from 0.38% citrated blood. After incubation with 10 μg/mL of mAb SZ-123, human IgG, or different concentrations of MHCSZ-123 at 37 °C for 10 min, platelet aggregation was induced by 1.25 mg/mL ristocetin (Sigma) under constant stirring (1000 rpm). Platelet aggregation was measured according to the change in light transmission using a lumiaggregometer (Chrono-log, Havertown, PA, USA).

### Effect of MHCSZ-123 on platelet adhesion under flow conditions

To examine whether MHCSZ-123 could inhibit platelet adhesion to collagen under physiological flow conditions, a flow chamber assay was performed. The effects of the antibody were tested under different shear rates of 1200 s^−1^ and 300 s^−1^ in a parallel-plate flow chamber (GlycoTech Inc., Gaithersburg, MD, USA). Acid-soluble collagen type III (200 μg/ml) from human placenta (Sigma) was coated onto dishes (35 × 10 mm, Corning, New York, NY, USA) at 4 °C overnight. Non-specific binding sites were blocked with 2% BSA-PBS. Whole blood from healthy volunteers was collected (low-molecular-weight heparin as anticoagulant, 40 U/ml), and platelets were labeled with calcein acetoxymethyl ester (calcein-AM, Dojindo Molecular Technologies, Rockville, MD, USA). After incubation with 10 μg/mL mAb SZ-123, SZ-34, or MHCSZ-123 for 10 min at 37 °C, perfusions were carried out at 37 °C in a water bath. During the 5-min perfusion, images were captured using a fluorescence microscope (Leica DMIL-LED, Wetzlar, Germany) at 0, 1, 3, and 5 min, respectively.

### Model of arterial thrombosis in Rhesus monkeys

Housing, treatment, surgery, and veterinary care for the animals used in the study were approved by the Animal Ethics Committee of Soochow University. Twenty-four Rhesus monkeys obtained from Xishan Zhongke Laboratory Animal Co., (Suzhou, China) were used in this study. The monkeys weighed between 4.0 and 6.5 kg and were aged 3–5 years old. Monkeys were distributed into four treatment groups according sex and weight. Groups 1–4 were administered saline, 0.1, 0.3, and 0.6 mg/kg MHCSZ-123, respectively. Each group contained six animals, half male and half female. Each animal underwent the experiment one time.

Arterial thrombosis was established in the monkeys as described previously [23, 27]. Briefly, monkeys were anesthetized by intravenous injection of 3% sodium pentobarbital (30 mg/kg). Body temperature was maintained at 37 °C. An incision was made on the leg to expose 3–4 cm of the femoral artery. Blood flow was measured by a Silastic tubing transducer (Triton Technology, San Diego, CA, USA) placed around the dissected artery. After a latency phase of 30 min to stabilize the flow, the endothelium of the femoral artery was injured for 20 s using three artery clamps with spring devices at intervals of 2 mm to damage the vascular wall. Next, arterial stenosis was applied by adjusting a plastic C-shaped constrictor placed over the injury site. Subsequently, a Fogarty balloon catheter (3.0–10 mm, Cordis Corporation International, Freemont, CA, USA) was inserted into the clearance between the C-shaped ring and the femoral artery. The balloon was inflated, and pressure was maintained to narrow the artery by 65–80%. Injury and stenosis resulted in thrombus formation in the femoral artery, detected as a reduction in the flow. When blood flow was reduced to nearly zero, the thrombus was dislodged by releasing the balloon pressure. This repetitive pattern of decreasing blood flow after mechanical restoration is referred to as cyclic flow reductions (CFRs) [23, 27].

Baseline CFRs were recorded for 60 min (*t* = −60–0 min), 5 mL of the test agents (saline or MHCSZ-123) were administrated by intravenous injection within 1 min (*t* = 0 min), and monkeys were monitored up to 1 h after administration (*t* = +60 min). The antithrombotic effect of MHCSZ-123 was quantified by comparing the number of CFRs per hour before and after administration of MHCSZ-123. Blood samples were drawn at different time points for ex vivo analysis.

### Platelet count, bleeding time, and coagulation

Blood collection and bleeding time measurements were performed at 0, 5, 15, and 30 min; and 1, 2, 4, and 24 h after drug administration. Samples were collected in 0.38% (final concentration) trisodium citrate, and the platelet count was measured immediately using an automatic blood cell analyzer (SYSMEX, Hyogo, Japan). The template bleeding time was measured using bleeding time devices (International Technidyne Corporation, Edison, NJ, USA). The surface of the foreleg was shaved, and a pressure cuff was applied with constant inflation to 20 mmHg during measurements. A wound incision approximately 5 mm long and 1 mm deep was then carefully dabbed every 15 s with sterile filter paper. All measurements were performed once at each time point. Prothrombin time (PT) and activated partial thromboplastin time (APTT) were measured at 37 °C using an automatic coagulometer (Stago, Asnières sur Seine Cedex, France).

### Plasma VWF level

Plasma VWF levels were measured by ELISA. Briefly, 96-well microtiter plates were coated with monoclonal antibody anti-VWF SZ-29-IgG (5 mg/mL) overnight at 4 °C [28] and blocked with 2% bovine serum albumin in (BSA-PBS) for 2 h at 37 °C. Next, standard human plasma pool (mixed plasma from 20 individuals with different blood types, set as 10 μg/ml) with serial dilutions (1:20,1:50,1:100, 1:200, 1:500, 1:1000) and diluted test samples (1:100), all in 0.2% BSA-PBS, were added into duplicate wells and incubated for 2 h at 37 °C. Bound VWF was incubated with monoclonal anti-VWF-antibody SZ-34 labeled with HRP for 2 h at 37 °C and then detected using TMB. Plasma VWF levels were calculated from a standard curve obtained by adding 1:20 to 1:1000 dilutions of a human plasma pool containing 10 μg/mL human VWF to coated wells.

### VWF occupancy

VWF occupied by MHCSZ-123 was estimated using a competition ELISA assay with a fixed concentration of biotinylated SZ-123 (Bio-SZ-123) (made by our lab) in combination with streptavidin-HRP (Thermo). Briefly, 96-well microtiter plates were coated with a polyclonal anti-VWF-Ig solution (10 μg/well) overnight at 4 °C and blocked with 2% BSA-PBS at 37 °C for 2 h. A dilution series (1:2 in PBS) of the plasma samples (pre-warmed for 5 min at 37 °C) was added for 2 h at 37 °C. Samples containing 100% occupied VWF were obtained by adding a saturating amount of MHCSZ-123 (6 μg/mL) to each corresponding Rhesus monkey plasma sample. Bound MHCSZ-123 was detected by addition of a fixed concentration of biotinylated SZ-123 (Bio-SZ-123) for another 1 h. After washing, bound Bio-SZ-123 was combined with streptavidin-HRP for 1 h at 37 °C. Visualization and wash steps were performed as described above. The VWF occupancy of each sample was calculated as follows: (A450 nm sample saturated with MHCSZ-123/A450 nm sample) × 100%.

### Collagen binding assay

VWF-collagen binding assays were performed by ELISA. Briefly, 96-well microtiter plates were coated overnight at 4 °C with type III collagen from human placenta and blocked with 2% BSA-PBS. Rhesus monkey plasma was then added to the wells and bound VWF detected using rabbit anti-human VWF-HRP antibodies. Binding of VWF to collagen in plasma samples at each time point was compared to the binding of VWF in each blood sample taken at time zero (pre-sample, *t* = 0 min), which was set as 100%.

### Statistical analysis

Data were expressed as mean ± SD. Statistical analysis was performed using GraphPad Prism 5 (San Diego, CA) for Student’s *t* test (paired) and one-factor ANOVA followed by Fisher’s exact test. *P* < 0.05 was considered significant.

## Results

### Cloning of MHCSZ-123 VH and VL genes

**Fig. 1:**
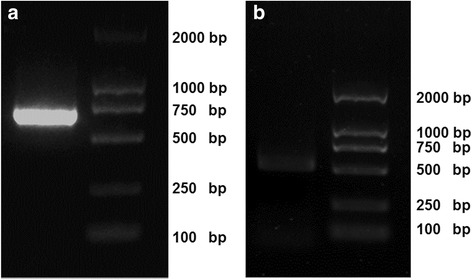
Cloning of monoclonal antibody SZ-123 variable sequences. Total RNA was extracted from hybridomas expressing SZ-123 monoclonal antibody. 5’RACE was performed and PCR products of VH (**a**) and VL (**b**) were separated in 1% agarose gels. The PCR products were 663 bp for VH and 543 bp for VL

We used 5’ RACE to clone the SZ-123 variable gene and the signal peptide sequence. The PCR products were 663 bp for VH and 543 bp for VL (Fig. 1). DNA sequencing and BLAST search against Kabat’s database confirmed that the PCR products were variable sequences of mouse monoclonal antibody. Both VH and VL contained sequences encoding 23- and 20-aa signal peptides, respectively, on their N-termini.

### Construction of the expression vectors pMH3-MHC-SZ-123VH and pMH3-MHC-SZ-123VL

**Fig. 2:**
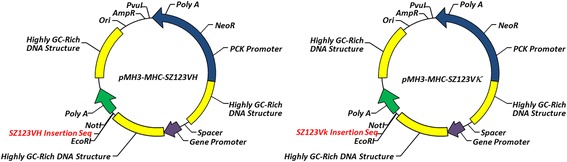
The mammalian expression vectors pMH3-MHCSZ-123VH and pMH3-MHCSZ-123-VK. Both vectors contain GC-rich promoter and chicken beta actin gene intron-1 followed by dhfr cDNA. pMH3-MHC-SZ-123VH contains the variable sequence of the SZ-123 mouse monoclonal antibody heavy chain and constant region sequence of the human IgG1 heavy chain. pMH3-MHC-SZ-123-VK contains the variable sequence of the SZ-123 mouse monoclonal antibody light chain and constant region sequence of the human kappa chain

To produce chimeric monoclonal antibody MHCSZ-123 effectively, we chose pMH3, which is an expression vector that contains ubiquitous chromatin opening elements (UCOEs), transfected into CHO-S cells as an expression system [26]. Detailed structures of pMH3-MHC-SZ-123VH and pMH3-MHC-SZ-123VL expression vectors are depicted in Fig. 2. The vectors contain a chicken β-actin promoter and three non-coding GC-rich regions. The cDNA fragment encoding MHC-SZ-123VH or MHC-SZ-123VL contains a native signal peptide. As shown in Fig. 2, the three “GC” elements (UCOEs) were located at the flanks of the promoter and the MHCSZ-123VH or MHCSZ-123VL fragment, respectively. The *Neo* gene was employed as a selection marker.

### Generation and identification of stable MHCSZ-123-expressing CHO-S cell clones producing high yields of antibody

More than 50 clones secreted MHCSZ-123 at higher levels than other clones, which were >12.5 mg/L as determined by ELISA. These clones were selected as high-expression cell clones and picked for the second round of selection. After two rounds of selection, five clones with the highest titers were selected under G418 for gene copy-number amplification. To select the cell clone that produced the highest level of antibody, the five chosen cell clones were adapted to suspension culture in serum-free medium and assays were performed on a small scale of 50 ml. The highest antibody-producing clone (23 μg/ml) was designated MHCSZ-123-CHO and chosen for further studies. We scaled the cultures of the highest producer clone up to 10 L for a fed-batch culture. MHCSZ-123 was collected and purified for further studies. As estimated using a Coomassie blue-stained sodium dodecyl sulfate polyacrylamide gel electrophoresis (SDS-PAGE) gel, MHCSZ-123 protein of >95% purity was obtained after affinity chromatography. The recovery rate of MHCSZ-123 reached 78.8% (63 mg/80 mg).

### MHCSZ-123 inhibited binding of Rhesus monkey VWF to collagen type III

**Fig. 3:**
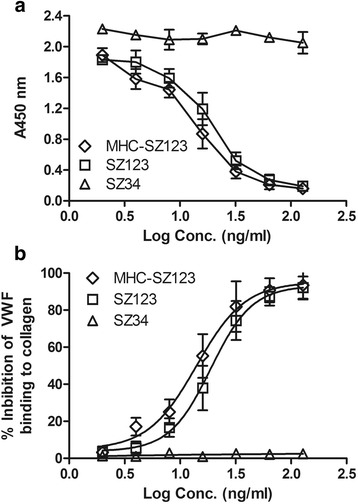
MHCSZ-123 blocked binding of Rhesus monkey VWF to human collagen type III. **a** Binding curve of Rhesus monkey VWF binding to collagen. **b** Inhibition of Rhesus monkey VWF to collagen. MHCSZ-123 and SZ-123 (2–128 μg/mL) were tested for blocking interactions between Rhesus monkey VWF and human type III collagen coated on plates. The percent inhibition was similar to the parent mAb SZ-123. mAb SZ-34, a non-blocking mAb to the human VWF A2 domain, was used as a negative control. Data are presented as mean ± SD (*n* = 6 animals)

As shown in Fig. 3, MHCSZ-123 inhibited binding of Rhesus monkey VWF to collagen type III in a dose-dependent manner in vitro, which was similar to the parent mAb SZ-123. A plasma concentration of 128 ng/mL caused approximately 100% inhibition of VWF binding to collagen. In contrast, negative control anti-VWF A2 mAb SZ-34 did not inhibit binding of VWF to collagen. The 50% inhibitory concentration (IC_50_) of MHCSZ-123 was 14.06 ± 1.34 ng/ml, which was slightly lower than that of the parent antibody SZ-123 (16.91 ± 2.07 ng/ml). However, the difference was not significant. We concluded that MHCSZ-123 cross-reacted efficiently with plasma VWF from Rhesus monkeys and specifically inhibited the binding of plasma VWF from Rhesus monkeys to human collagen type III. Thus, Rhesus monkeys are an appropriate model for investigating MHCSZ-123 as a potential therapeutic agent for arterial thrombosis.

### MHCSZ-123 inhibited ristocetin-induced platelet aggregation in vitro

**Fig. 4:**
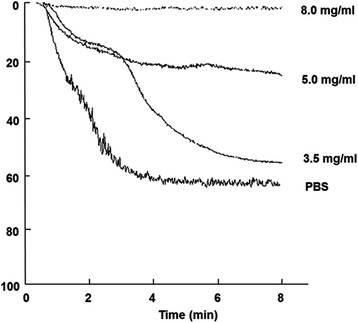
MHCSZ-123 inhibited ristocetin-induced platelet aggregation in a dose-dependent manner (3.5, 5, and 8 mg/mL of MHCSZ-123). Platelet aggregation was induced by ristocetin (1.25 mg/mL) under constant stirring conditions

The activity of MHCSZ-123 was further tested for its effect on ristocetin-induced platelet aggregation in vitro. Similar to murine SZ-123, purified MHCSZ-123 at a concentration of 8 μg/mL completely inhibited ristocetin-induced human platelet aggregation (Fig. 4).

### MHCSZ-123 inhibited human platelet adhesion to collagen under high shear stress

**Fig. 5:**
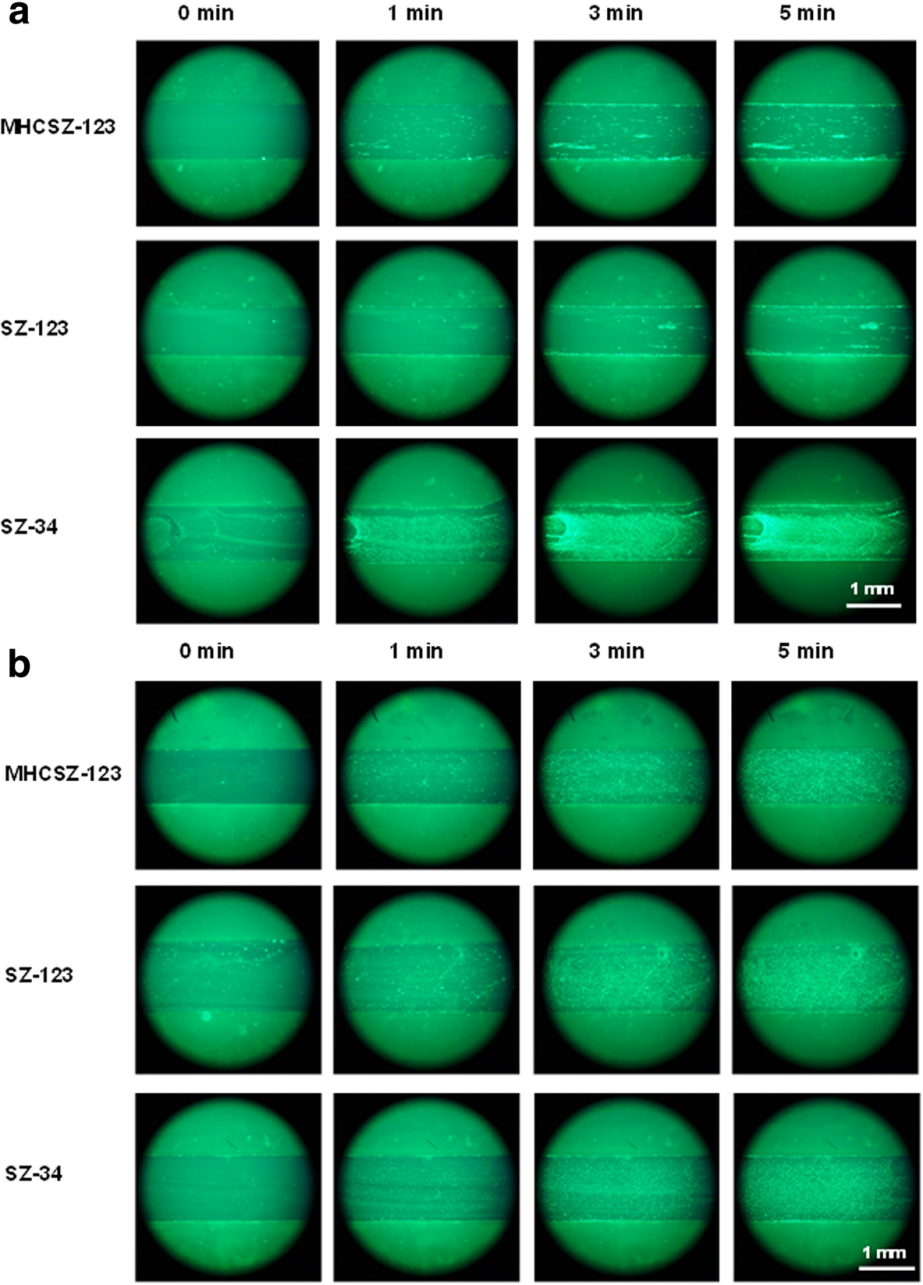
Inhibition of human platelet adhesion to collagen by MHCSZ-123 under shear stress. Heparinized whole blood was perfused over the collagen substrate at a constant high (1200 s^−1^) or low (300 s^−1^) shear rate at 37 °C for 5 min. The fluorescence microscope images of platelet adhesion at 0, 1, 3, and 5 min of perfusion under high or low shear stress are presented (10 × 20). **a** MHCSZ-123 inhibited VWF binding to collagen under high shear stress, similar to the parent mAb SZ-123. **b** Neither MHCSZ-123 nor SZ-123 inhibited VWF binding to collagen under low shear stress. The concentration of antibodies was 10 μg/ml. The non-inhibitory antibody SZ-34 (10 μg/ml) was used as a control

The perfusion chamber thrombosis model was used to evaluate whether the antibodies affected thrombus formation under different flow conditions. As shown in Fig. 5, MHCSZ-123 inhibited VWF binding to collagen under 1200 s^−1^ shear stress (Fig. 5a), which was similar to that observed in the parent mAb SZ-123. Neither MHCSZ-123 nor SZ-123 inhibited VWF binding to collagen under 300 s^−1^ shear stress (Fig. 5b).

### MHCSZ-123 inhibited platelet thrombosis in vivo

**Fig. 6:**
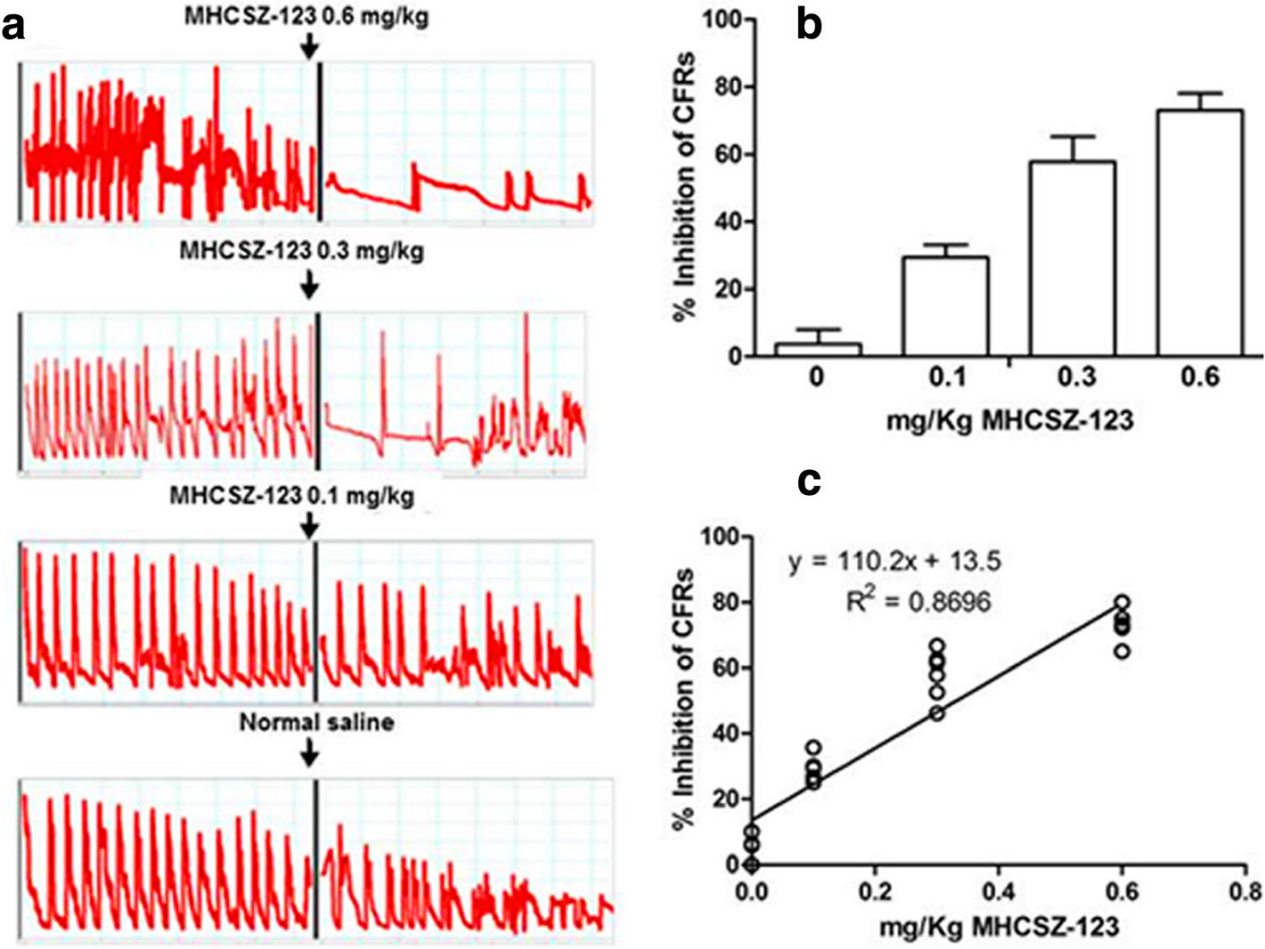
MHCSZ-123 inhibited high-shear arterial thrombosis in the Rhesus monkey model. **a** Representative charts of CFRs showing the effect of MHCSZ-123 on platelet thrombosis induced in the femoral arteries of Rhesus monkeys. Changes in CFRs were recorded from −60 to 60 min. *Arrows* indicate points at which MHCSZ-123 (0.1, 0.3, and 0.6 mg/kg MHCSZ-123) or saline was administered. **b** Inhibition of CFRs measured within 1 h after administration of different doses of MHCSZ-123 (0, 0.1, 0.3, and 0.6 mg/kg). **c** Correlation between MHCSZ-123 dose and inhibition of CFRs. Data represent the mean ± SD (*n* = 6 animals per group)

The in vivo antithrombotic effect of MHCSZ-123 was evaluated by administering different doses (0, 0.1, 0.3, and 0.6 mg/kg) of MHCSZ-123 to Rhesus monkeys in a modified arterial platelet thrombosis model. CFRs were significantly inhibited (*P* < 0.01) by administration of MHCSZ-123 at 0.1, 0.3, and 0.6 mg/kg (Fig. 6a). A high dose of MHCSZ-123 (0.6 mg/kg) inhibited CFRs by 73.1 ± 4.9% (Fig. 6a); a medium dose (0.3 mg/kg) of MHCSZ-123 inhibited CFRs by 57.9 ± 7.4%; and a low dose (0.1 mg/kg) of SZ-123 inhibited CFRs by 29.4 ± 3.7% (Fig. 6b). There was a good agreement between CFR inhibition and MHCSZ-123 dose (*P* < 0.01; Fig. 6c).

### Occupancy of VWF binding sites by MHCSZ-123

**Fig. 7:**
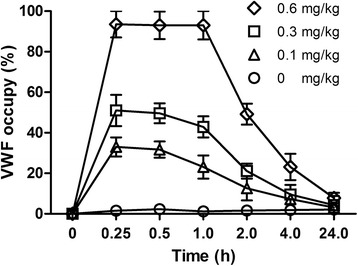
VWF occupancy after administration of MHCSZ-123. At 15 min after injection, the mean VWF occupancy was 33% at 0.1 mg/kg, 51% at 0.3 mg/kg, and 94% at 0.6 mg/kg, respectively. The binding was stable by 1 h after injection. At 2 h after administration, occupancy was still observed. Data represent mean ± SD (*n* = 6 animals per group)

At 15 min after administration, the VWF occupancy was 33.5 ± 6.5% at 0.1 mg/kg, 51.0 ± 7.7% at 0.3 mg/kg, and 93.5 ± 6.5% at 0.6 mg/kg, respectively. The binding was stable by 1 h after injection. At 2 h after administration, occupancy was still observed. At 24 h after administration, binding was no longer observed (Fig. 7).

### MHCSZ-123 inhibited VWF binding to collagen ex vivo

**Fig. 8:**
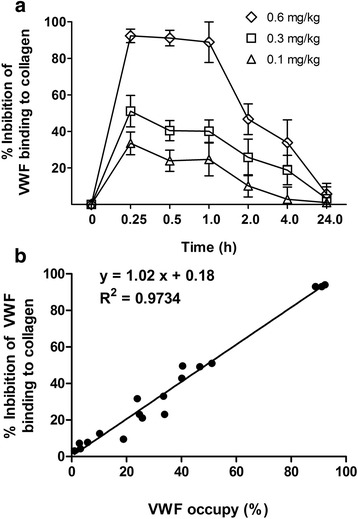
Ex vivo inhibition of the binding of Rhesus monkey VWF to collagen by MHCSZ-123. **a** Ex vivo inhibition of Rhesus monkey VWF binding to collagen by MHCSZ-123. **b** Correlation between ex vivo VWF occupancy and inhibition of VWF binding to collagen. Data represent mean ± SD (*n* = 6 animals per group)

The ability of MHCSZ-123 to inhibit the binding of Rhesus monkey VWF to collagen was evaluated at different time points after MHCSZ-123 administration. The binding of Rhesus monkey VWF to collagen was significantly inhibited 15 min after administration, and the inhibition effect was stable by 1 h after injection. At 2 h after administration, inhibition was still present. The inhibitory effect was gone by 24 h (Fig. 8a). There was a good agreement between the results of ex vivo VWF occupancy and the inhibition of VWF binding to collagen (Fig. 8b).

### MHCSZ-123 inhibited VWF-dependent platelet aggregation

**Fig. 9:**
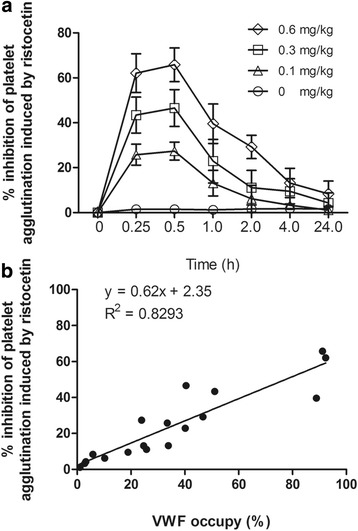
MHCSZ-123 inhibited VWF-dependent platelet aggregation. **a** Ex vivo inhibition of MHCSZ-123 on ristocetin-induced Rhesus monkey platelet aggregation. **b** Correlation between ex vivo VWF occupancy and inhibition of platelet aggregation induced by ristocetin. Data represent mean ± SD (*n* = 6 animals per group)

MHCSZ-123 significantly inhibited ex vivo ristocetin-induced platelet aggregation at 0.1, 0.3, and 0.6 mg/kg (Fig. 9a). The inhibition of ristocetin-induced platelet aggregation by 0.6, 0.3, and 0.1 mg/kg MHCSZ-123 was maximal at 0.25 h after injection, where the maximum inhibition was 62.1 ± 8.6%, 43.4 ± 8.0%, and 25.8 ± 4.7%, respectively. The inhibitory effect of MHCSZ-123 on platelet aggregation was gone by 24 h (Fig. 9a).

We also observed a correlation between ex vivo VWF occupancy and inhibition of ristocetin-induced platelet aggregation (Fig. 9b). Occupation of 80% of circulating VWF caused a 50% inhibition of ristocetin-induced platelet aggregation.

### MHCSZ-123 did not affect VWF levels, platelet count, bleeding time, or coagulation in vivo

**Fig. 10:**
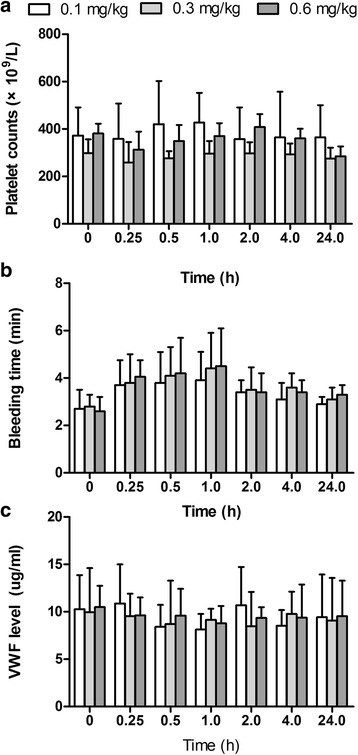
MHCSZ-123 did not affect platelet count, bleeding time, or VWF levels in plasma. Peripheral platelet count (**a**), bleeding time (**b**), and VWF levels in plasma (**c**) at different time points after administration of varying doses of MHCSZ-123. Data represent mean ± SD (*n* = 6 animals per group). No significant differences were observed between doses

No significant changes in platelet count were observed in animals after treatment with MHCSZ-123 when compared with the *t* = 0 injection of MHCSZ-123 (Fig. 10a). Bleeding time was not significantly prolonged after injection of 0.1, 0.3, and 0.6 mg/kg MHCSZ-123 (Fig. 10b). Plasma VWF antigen levels were not affected by treatment and no significant changes were observed between pre- and post-treatment of Rhesus monkeys with MHCSZ-123 (Fig. 10c).

In addition, there were no significant changes in coagulation parameters (PT and APTT) observed in any of the groups (data not shown). All animals survived after the procedures, and none exhibited bleeding complications.

## Discussion

VWF initiates thrombus formation by mediating platelet adhesion to exposed subendothelial collagen on an injured vessel wall. It is known that bridge formation between platelets and collagen by VWF allows platelets to withstand the elevated shear forces, whereas the role of VWF at lower shear forces is redundant at best. Thus, interfering with this adhesion pathway might allow specific targeting of the antithrombotic action to stenotic arteries, leaving the rest of the circulation intact, with presumably less risk for spontaneous bleeding [1].

We showed that the chimeric monoclonal anti-VWF-A3 antibody, MHCSZ-123, inhibited platelet adhesion to human collagen type III in a perfusion flow chamber; this effect was more efficient with increasing shear rate as observed with the parent mAb SZ-123 [22]. The antithrombotic property of the mAb was demonstrated in a Rhesus monkey model of arterial thrombosis [23] in which intravenous injection of SZ-123 at 0.6 mg/kg nearly completely blocked platelet-dependent thrombus formation. This antithrombotic effect was not accompanied by a prolongation of the bleeding time, which is in contrast to what is normally found with regular antiplatelet drugs [4, 5]. In view of these promising results, we recently prepared a mouse/human chimeric version of SZ-123, MHCSZ-123, with similar affinity and in vitro and ex vivo efficacy as the murine SZ-123 in different models.

The stable expression system we used allowed high yield and quality of recombinant protein in different batches for further research. The HEK293 and CHO cell lines are the most frequently used mammalian cells for protein expression because they produce proteins with correct folding and post-translational modifications [29, 30]. The CHO cell line is widely used for stable production of proteins at large scale and high density. Many pharmaceuticals have been produced in CHO cells [31]. Therefore, we expressed MHCSZ-123 using the CHO cell line. For efficient production of recombinant protein, the expression vector is essential in addition to the host cell. pMH3 is a eukaryotic expression vector (with GC-rich UCOEs) that is suitable for the CHO expression system [26]. The three non-coding UCOEs in the vector were designed and modified to increase the transcriptional activity of exogenous genes. Therefore, the pMH3 vector protects the insert of interest from silencing and leads to highly efficient and stable production of proteins no matter where their integration sites are [26].

In the present study, we demonstrated again that MHCSZ-123 inhibited VWF binding to collagen ex vivo in a dose- and time-dependent manner. As we previously observed with the murine SZ-123, 60% of VWF occupied by SZ-123 is enough to totally inhibit the thrombus formation but also to inhibit the ex vivo platelet aggregation induced by 1.25 mg/ml ristocetin [22]. Kageyama et al. [11] showed that 50% VWF occupancy by AJW200 was enough to completely inhibit CFRs and botrocetin-induced platelet aggregation ex vivo. Nevertheless, it is clear that the actual receptor occupancy likely might be underestimated because biotinylated SZ-123, used to determine the remaining free binding sites, will to some extent compete with the bound MHCSZ-123.

## Conclusions

In conclusion, the present study demonstrated that the chimeric monoclonal antibody MHCSZ-123 was a powerful inhibitor of the GPIbα-VWF-collagen axis. In addition, we confirmed that inhibiting the platelet VWF-collagen interaction is an efficient way to prevent thrombus formation in the injured and stenosed Rhesus monkey femoral artery without causing prolongation of the bleeding time or increased blood loss. Because of the lack of effect on the bleeding time, it will be worthwhile to validate this approach in clinical trials aimed to prevent acute arterial thrombotic syndromes.

## Abbreviations

5’RACE: 5’ Rapid amplification of cDNA ends
APTT: Activated partial thromboplastin time
BSA: Bovine serum albumin
CFRs: Cyclic flow reductions
CHO-S: Chinese hamster ovary-S
ELISA: Enzyme-linked immunosorbent assay
GP: Glycoprotein
HRP: Horseradish peroxide
mAb: Monoclonal antibody
PBS: Phosphate-buffered saline
PPP: Platelet-poor plasma
PRP: Platelet-rich plasma
PT: Prothrombin time
SDS-PAGE: Sodium dodecyl sulfate polyacrylamide gel electrophoresis
TMB: 3,3’,5,5’-Tetramethylbenzidine
UCOEs: Ubiquitous chromatin opening elements
VWF: Von Willebrand factor
